# Isolation and *in vitro* characterization of novel *S. epidermidis* phages for therapeutic applications

**DOI:** 10.3389/fcimb.2023.1169135

**Published:** 2023-05-24

**Authors:** Vida Štrancar, Monika Marušić, Jasmina Tušar, Neža Praček, Marko Kolenc, Katja Šuster, Simon Horvat, Nika Janež, Matjaž Peterka

**Affiliations:** ^1^ Centre of Excellence for Biosensors, Instrumentation and Process Control, Ajdovščina, Slovenia; ^2^ Department of Animal Science, Biotechnical Faculty, University of Ljubljana, Domžale, Slovenia; ^3^ Institute of Microbiology and Immunology, Faculty of Medicine, University of Ljubljana, Ljubljana, Slovenia; ^4^ Valdoltra Orthopaedic Hospital, Ankaran, Slovenia

**Keywords:** therapeutic phages, phage safety and efficacy, prosthetic joint infections, biofilms, *Staphylococcus epidemidis*

## Abstract

*S. epidermidis* is an important opportunistic pathogen causing chronic prosthetic joint infections associated with biofilm growth. Increased tolerance to antibiotic therapy often requires prolonged treatment or revision surgery. Phage therapy is currently used as compassionate use therapy and continues to be evaluated for its viability as adjunctive therapy to antibiotic treatment or as an alternative treatment for infections caused by *S. epidermidis* to prevent relapses. In the present study, we report the isolation and *in vitro* characterization of three novel lytic *S. epidermidis* phages. Their genome content analysis indicated the absence of antibiotic resistance genes and virulence factors. Detailed investigation of the phage preparation indicated the absence of any prophage-related contamination and demonstrated the importance of selecting appropriate hosts for phage development from the outset. The isolated phages infect a high proportion of clinically relevant *S. epidermidis* strains and several other coagulase-negative species growing both in planktonic culture and as a biofilm. Clinical strains differing in their biofilm phenotype and antibiotic resistance profile were selected to further identify possible mechanisms behind increased tolerance to isolated phages.

## 1 Introduction

The coagulase-negative bacterium *Staphylococcus epidermidis* is a common colonizer of human skin. However, once it crosses the epithelial barrier, it can cause infections of implanted medical devices, such as artificial joints (Otto, 2009). The main cause of prosthetic joint infections are coagulase-negative staphylococci (CoNS), with *S. epidermidis* being the most common pathogen (Zimmerli, 2014; Van Epps and Younger, 2016; Patel, 2023). These infections are chronic because *S. epidermidis* has excellent ability to adhere to biomaterials and form biofilms (Rupp and Archer, 1994; Darouiche, 2004; Wisplinghoff et al., 2004). Therefore, complex treatment protocols with prolonged antimicrobial therapy and multiple surgeries are required in 20% of cases of prosthetic joint infections (Zimmerli, 2014; Kandel et al., 2019). In the event of treatment failure, there are limited treatment strategies to prevent recurrence (Zimmerli, 2014).

Phage therapy is already being used to treat sporadic cases of orthopedic infections as part of compassionate use (expanded access therapy) and clinical trials are underway in the United States and France to gain further evidence of the efficacy of phage therapy (Onsea et al., 2019; Ferry et al., 2020; Doub et al., 2021). The advantages of virulent phages as therapeutic agents are their narrow specificity, their ability to replicate in the presence of a suitable host, and their potential anti-biofilm activity (Loc-Carrillo and Abedon, 2011). Although guidelines for phage therapy are still being developed, a widespread agreement on the features of therapeutic phages has been accepted. Therapeutic phages need to be strictly virulent, without functional genetic determinants of the lysogenic cycle, virulence factors, or antibiotic resistance genes, and should not be prone to generalized transduction (Pirnay et al., 2015). Many of these features are addressed at the level of the whole genome sequence, which is accepted as fundamental information about therapeutic phages (FDA, 2021; Center for Biologics evaluation and research, and National Institute of Allergy and Infectious Disease, 2021). Prior to any analysis of genome content, a high-quality whole genome sequence must be obtained (Turner et al., 2021). Currently, open-access tools are available that can be used to develop a workflow to generate the required data and allow for easy repetition (Shen and Millard, 2021). Genome content analysis is based on similarity searches between coding phage sequences and quality databases, including virulence factors and genetic determinants of antibiotic resistance. However, genetic indications of phage lifestyle and lack of general transduction must be supported also by experimental evidence. Therapeutic phages also need to be active against target pathogens with diverse backgrounds (broad host range) without affecting the accompanying microbiota to minimize side effects (Pirnay et al., 2015). Phages that meet these criteria are then extensively tested for safety and efficacy in clinical trials prior to any therapeutic application. Phage preparations for clinical trials must comply with good manufacturing practice guidelines established by regulatory agencies (EMA, FDA) to eliminate the possibility of contamination and errors (Pelfrene et al., 2016; Plaut and Stibitz, 2019). The criteria of identity and purity must be met to ensure that phages retained their characteristics during the production and that the phage preparations are free of potentially harmful contaminants (exotoxins, endotoxins, host cell proteins, host cell DNA) (Merabishvili et al., 2009).

*Staphylococcus* specific phages with demonstrated therapeutic potential from the genus *Kayvirus*, ISP, K, Sb-1, vB_SauM-fRuSau02, SaGU1 and phages comprising the clinically tested cocktail AB-SA01 (J-Sa36, Sa38 and Sa87) are polyvalent and highly effective against *S. aureus*, but their infectivity against coagulase-negative species is either reduced or underexplored (O’Flaherty et al., 2005; Kvachadze et al., 2011; Vandersteegen et al., 2013; Leskinen et al., 2017; Fish et al., 2018; Lehman et al., 2019; Kifelew et al., 2020; Shimamori et al., 2021). Currently known *S. epidermidis*-specific virulent phages belong to the siphovirus, podovirus, and myovirus morphotypes, with the latter two being strictly virulent (Melo et al., 2014; Gutiérrez et al., 2015; Cater et al., 2017; Valente et al., 2021; Fanaei Pirlar et al., 2022). Most information is available on the SEP1 and IPLA-C1C phages, which belong to the *Sepunavirus* genus and have high specificity for *S. epidermidis*, but decreased infectivity toward *S. aureus* (Melo et al., 2014; Gutiérrez et al., 2015a). Despite its ability to infect bacteria in different physiological states, phage SEP1 is unable to efficiently clear biofilms of *S. epidermidis* (Melo et al., 2018; Melo et al., 2020). Similarly, poor efficacy against biofilms was previously reported for *Staphylococcus* phage K, despite its activity against planktonic cultures, suggesting that some virulent phages may have limited anti-biofilm activity (Cerca et al., 2007; Alves et al., 2014). Conversely, the phage IPLA-C1C has been shown to have biofilm-dispersing activity (Gutiérrez et al., 2015).

Intrinsic resistance mechanisms to phages in *Staphylococcus* are attributed to widespread and well-studied restriction-modification systems (RMS), particularly type I, II and IV (Moller et al., 2019). In addition, superinfection immunity, assembly interference, and abortive infection may affect the phage host range, whereas the presence of CRISPR systems in staphylococci is limited (Moller et al., 2019). Resistance acquired by spontaneous mutations has not been described in detail in *S. epidermidis* (Jurado et al., 2022). However, in response of *S. aureus* to a nonlethal dose of the phage phiIPLA-RODI, the synthesis of DNA-rich biofilms has been observed, which help the bacteria resist environmental stress, while also maintaining a reservoir of dormant susceptible bacteria that would support phage replication if reactivated (Fernández et al., 2017). The acquisition of phage resistance can be delayed to some extent by rational phage cocktail design, that includes phages that infect bacteria with different phage resistance mechanisms (Melo et al., 2014; Lehman et al., 2019). There is also growing evidence that resistance acquired during phage treatment is associated with reduced fitness of the resistant strain, e.g., reduced colonization capacity, thus making them more susceptible to conventional therapies (Mangalea and Duerkop, 2020).

This work reports the isolation and *in vitro* characterization of three novel *S. epidermidis* phages that exhibit broad activity against a range of clinical strains, and are lytic, non-transducing and do not carry non-desirable genes making them suitable candidates for further *in vivo* safety and efficacy studies. We also demonstrate the importance of making informed decisions regarding isolation and propagation strains already at the early stage of phage research. We address several aspects of efficacy testing and highlight potential resistance mechanisms of clinical strains with respect to phage efficacy.

## 2 Materials and methods

### 2.1 Bacterial strains and growth conditions

The origin and characteristics of 44 *S. epidermidis* and 12 *S. aureus* strains are shown in **Figure S1**, along with additionally tested *S. pseudintermedius, S. pasteuri*, *S. lugdunensis*, *S. capitis* and *S. pettenkoferi* strains. *Staphylococcus* was cultivated on tryptone soya broth (TSB, Sigma-Aldrich, USA) agar (Biolife, Italy) plates or broth at 37°C and 150 rpm. For the double agar overlay assay TSB was supplemented with 1.2% agar to obtain agar plates or with 0.4% agar for soft agar overlay.

### 2.2 Phage isolation, propagation, and quantification

Phages were isolated from local wastewater treatment plants in Slovenia between December 2020 to July 2021 (**Table S1**). Collected influent composite samples (24 h) or influent grab samples were centrifuged and then filtered through 0.45 µm and 0.22 µm PES membrane filters (TPP). Ten-times concentrated (10x) TSB was added to sewage to obtain 1x TSB. The medium was supplemented with magnesium sulphate (10 mM) and calcium chloride (1 mM) and inoculated with 1% of the overnight culture, followed by incubation at 37°C and 150 rpm. Strains used for enrichment are listed in **Table S2**. The enrichment mixtures consisted of only one bacterial host at a time. Individual sewage samples were enriched separately on multiple hosts. For detection of phages, the overnight enrichment cultures were centrifuged and the supernatants were spotted on bacterial lawns of different strains following protocol for spot assay (Carlson, 2005). Clarification of the indicator strain indicated the presence of phages and these lysis zones were excised and resuspended in SM buffer (50 mM Tris-Cl, pH 7.5, 100 mM NaCl, 8 mM MgSO_4_, 0.01% (w/v) gelatin). Lysis zones from enrichments of the same initial environmental sample produced on bacterial lawns of multiple hosts were merged in some cases (indicated in **Table S2**). Single plaques were obtained from these zones by double agar overlay technique (Kropinski et al., 2009). Pure phage population was obtained by three consecutive single plaque purifications using the double agar overlay technique.

Pure phage isolates were propagated via plate lysate as described elsewhere (Bonilla et al., 2016). For routine quantification, the double agar overlay technique was used. Briefly, 100 μL serial dilutions of the phage lysate were mixed with 100 μL of the propagation strain and was added to TSB overlay agar, which was poured onto TSB agar plates. After overnight incubation, plates with 30–300 plaques were counted, and phage titer was calculated. Phages were named with prefixes CO (research institution name abbreviation) and P (phage) followed by unique identifier as suffix. The names of all isolates are listed in **Table S2**.

### 2.3 Transmission electron microscopy

Formvar-coated grids were placed on drops of different phage suspensions for 5 min and negative staining was performed using 2% phosphotungstic acid (PTA). If needed, phage suspensions were ultracentrifuged using the Beckmann Coulter Airfuge® (Brea, USA) at high speed (100,000 x g) to concentrate phages directly on the grids and negative staining was performed by the same procedure. The grids were examined using a transmission electron microscope JEOL JEM-1400 Plus (Tokyo, Japan) at 120 kV.

### 2.4 Host range and efficiency of plating

Host range and EOP were determined as previously described (Kutter, 2009). Briefly, 10 µl of each phage tested at a concentration of 10^8^ PFU/ml was spotted onto the overlay agar of each bacterial strain, plates were then incubated at 37°C for 16-18 h. The degree of lysis was determined visually the following day (**Figure S1**). EOP for each strain was calculated as the titer of the tested strain divided by the titer on the phage propagation strain COB-Sec2.

### 2.5 Phage genome sequencing and analysis

Phage DNA was extracted from DNAse-treated phage lysates using the Phage DNA Isolation Kit (Norgen, Canada) according to the modified protocol from the manufacturer. Phage lysates were treated with 10 U/ml saltonase (Blirt, Poland) at room temperature and were heat inactivated after overnight incubation in the presence of DTT. The absence of host DNA was confirmed using the 16S rRNA-specific primer set fD1 and RU1406 before extraction (Olsen et al., 1986; Weisburg et al., 1991). Host DNA-free lysate was treated with proteinase K (20 mg/ml) at 55°C, for 30 min, followed by heat inactivation. Extraction was then performed according to the manufacturer’s instructions. The quality of the phage genomic DNA was determined by agarose gel electrophoresis. Sequencing libraries were prepared using the Nextera XTL library preparation kit (Illumina), and sequencing was performed using MiSeq or NovaSeq (Illumina) from a commercial vendor. Quality read trimming and removal of adapters was performed using Trimmomatic (Bolger et al., 2014). Spades or Unicycler were used to initially assemble phage genomes that resulted in one or more potential phage contigs and a depth of coverage greater than 2500x, as indicated by read mapping statistics performed by bwa (**Table S3**) (Li, 2013; Wick et al., 2017; Prjibelski et al., 2020). Therefore, subsampling was performed using seqtk to achieve 100x coverage^1^. The subsampled reads were assembled using Spades (Prjibelski et al., 2020). Quality trimmed reads and subsampled reads were mapped to these assemblies. Unmapped reads were extracted from the bam file and processed as described below. Sequenced phages that were free of contamination were assembled as single contig of the same size, regardless of the number of reads used for assembly, and subjected to further analysis. Preliminary identification of the closest relatives was performed by a stand-alone *blastn* against the Inphared dataset (Cook et al., 2021). The genomes of the most significant *blastn* hits were compared to the phage contigs using the ANI calculator via the web server (**Table S5**)^2^. The nature of genome termini was predicted from comparative analysis of the large terminase subunits from phages with experimentally determined packaging type as described by Merrill et al. (2016). Phage contigs were then co-linearized with the genomes of the ICTV exemplar isolates based on Mauve alignment. Annotation was performed with Prokka, along with functional annotation using the PHROGs database and transfer RNA (tRNA) and non-coding RNA (ncRNA) detection (Terzian et al., 2021). Structural predictions and domain searches were performed using InterProScan (Zdobnov and Apweiler, 2001). Indications of temperate lifestyle were firstly identified by *blastp* against lysogeny associated proteins (integrase, excisionase, recombinase, transposase, ParB) extracted from Inphared database and *blastn* against the NCBI non-redundant nucleotide database^3^. Phage genomes were also analyzed by PHACTS (McNair et al., 2012) to predict their lifestyle and Phaster to detect att sites (Arndt et al., 2016). Annotated coding sequences of phage contigs were analyzed with VFAnalyzer (Liu et al., 2019) and *blastp* against its database to detect any virulence factors, and with CARD web server to identify antibiotic resistance genes (McArthur et al., 2013). Quality trimmed phage reads were also mapped to the genome of the propagation organism, and by calculating mapping statistics (number of mapped reads, their distribution among contigs, coverage and depth) further indications of generalized transduction were obtained.

### 2.6 Phage contamination detection

Quality trimmed reads that did not map to phage contig were extracted from bam file using samtools and were assembled by Spades (Danecek et al., 2021). Obtained assemblies were analyzed by *blastn* against Inphared database to detect any prophage contamination. Contigs larger than 1000 bp were investigated also by *blastn* against NCBI non-redundant nucleotide database.

### 2.7 Dynamic host range

The growth kinetics was determined for phages free from prophage contamination, COP-80A, COP-80B and COP-110 and their selected hosts by measuring optical density (OD_600_). A freshly prepared bacterial culture with an OD_600_ of 0.25 (corresponding to 1.2×10^7^ CFU/ml) was diluted 1:10 in TSB and aliquoted onto a 96-well plate. The phage lysates were first diluted into working stock using TSB and then serially diluted to obtain MOIs of 0.1, 1, 10 and 100. After adding the phage dilutions to the bacterial aliquots, the optical density (OD_600_) was measured every 30 min for 24 h at 37°C with orbital shaking using the Synergy H1 microplate reader (Biotek, USA). Bacterial concentration (CFU/ml) was determined for bacterial inoculum along with phage titer to verify MOI. The experiment was performed in two biological replicates with four technical replicates per experiment. All experiments included a bacterial growth control and a negative control being TSB and TSB with phage stock. Data was plotted using Orange (Demšar et al., 2013).

Endpoint OD_600_ values were defined as OD_600_ measurements at time 24 h for every monitored bacterial culture. Duration of growth inhibition for each tested bacterial culture was determined as the time interval when OD_600_ values were below 0.1. Lag phase of 1.5 hours at the start of the measurement was excluded from inhibition time calculation, thus the maximum possible inhibition time was 22.5 h.

### 2.8 Statistical analysis

All experiments were performed independently two times with four technical replicates. Statistical significance of the experimental results (significance level of p<0.05) was calculated using unpaired two-sided t-test assuming unequal variances using Microsoft Excel. Data were compared pairwise among treated group and corresponding control.

### 2.9 Brightfield microscopy

*Staphylococcus* biofilms were prepared on 96-well PS, µCLEAR, high binding microtiter plates (Greiner Bio-One, Austria). Each well was inoculated with exponentially growing bacteria at an OD_600_ of 0.5 and incubated at 37°C for 24 h. Prior to exposure to phage bacterial cultures were aspirated and wells were washed twice with 1x PBS. The wells were then filled with phage lysates prepared by mixing phage lysate in SM buffer with 2x TSB at a ratio of 1:1. The added phage titers corresponded to an approximate MOI of 10. The non-treated control solution was prepared by mixing SM buffer and 2x TSB in a 1:1 ratio. The biofilms were treated with phages for 24 h at 37°C. Biofilms were visualized with an inverted microscope (Olympus, Japan) at 100× magnification with oil immersion. Captured images were then processed in ImageJ (Schneider et al., 2012). Brightness and contrast of each image were adjusted to enhance biofilm features. Each combination of biofilm and phage was tested in five replicates.

### 2.10 Antibiotic susceptibility testing

Antibiotic sensitivity of *S. epidermidis* was determined with Sensititre plate adapted for genus *Staphylococcus* (EUSTAPH AST plate, Thermo Fisher) according to manufacturer’s instructions.

### 2.11 Crystal violet assay

Biofilm phenotype of selected *S. epidermidis* strains (**Table 1**) was determined by crystal violet (CV) staining of the biofilm biomass, as previously described (Kwasny and Opperman, 2010). Briefly, overnight cultures of the strains tested were diluted 100-fold in TSB and inoculated into sterile 96-well assay plates with either hydrophobic (Eppendorf, Germany) or hydrophilic surfaces (TPP, Switzerland). After 16-18 hours of incubation, the plates were washed three times with deionized water. The washed biofilms were fixed by incubation at 60°C for 60 min. Then, biofilms were stained with 0.06% (w/v) CV for 15 min. Excess CV was washed three times with deionized water, remaining stain was then resuspended in 30% acetic acid and absorbance at 600 nm (A_600_) was measured. Biofilm assays were repeated at least twice on separate days, with four technical replicates assessed each time. Two type strains were used as reference strains, RP62A representing a biofilm proficient strain and ATCC 12228 a biofilm-negative strain. The biofilm phenotype was graded in comparison to the measurements in the control strains: strong (A_600_ ≥ A_600_(RP62A)), moderate (A_600_(ATCC 12228) < A_600_ < A_600_(RP62A)) or weak (A_600_ ≤ A_600_(ATCC 12228)).

**Table 1:** Biofilm phenotype, antibiotic resistance profile, and phage susceptibility of selected *S. epidermidis* strains.

strain	biofilm phenotype		antibiotic resistance	efficiency of plating (EOP)		
/	hydrophilic surface	hydrophobic surface	/	COP-80A	COP-80B	COP-110
COB-Sec1	weak	weak	clindamycin, tetracycline	0.873	2.193	1.027
COB-Sec2	moderate	moderate	clindamycin	1.000	1.000	1.000
COB-SE3	strong	moderate	erythromycin, tetracycline	1.057	1.269	1.061
COB-SE6	strong	weak	clindamycin, erythromycin, gentamicin*, levofloxacin*, moxifloxacin, tetracycline, tobramycin*, trimethoprim/sulfamethoxazole*	0	0	0
COB-SE8	moderate	moderate	cefoxitin, clindamycin*, erythromycin*, gentamicin*, moxifloxacin, tobramycin*, trimethoprim/sulfamethoxazole*	0.011	0.018	0.012
COB-SE11	strong	weak	tetracycline	0	0	0

^*^ Genetic determinant for antibiotic resistance found in strain’s genome. Susceptibility of selected strains to isolated phages is given as efficiency of plating (EOP) which is the titer of the phage on a given bacterial strain compared to the titer on the phage propagation strain (COB-Sec2 in this study). COB-Sec1 and COB-Sec2 were the most susceptible strains to all isolated phages (**Figure S1**), serving as isolation strains and propagation hosts (**Table S2**). COB-SE3 and COB-SE8 are clinical isolates susceptible to isolated phages, but have different clinical and genetic background. Another two clinical strains, COB-SE6 and COB-SE11, are resistant to phages and encode complete prophages and other potential phage resistance mechanisms.

### 2.12 Bacterial genome sequencing and analysis

*S. epidermidis* DNA was extracted using the High Pure PCR Template Preparation Kit (Roche, Switzerland). Briefly, overnight cultures were harvested by centrifugation and the pellet was treated with 0.05 mg lysozyme and 2.5 µg RNAse for 2 h in PBS. Then, 200 µl of binding buffer and 40 µl of proteinase K (supplied by the manufacturer) were added, and the mixture was incubated at 55°C for 30 min and then at 75°C for 10 min. Further purification steps were performed according to the manufacturer’s instructions. Paired-end libraries were generated using the TruSeq DNA Nano Library Preparation Kit (Illumina), followed by sequencing using a commercial vendor’s NovaSeq (Illumina). Trimming of quality reads and removal of adapters was performed using Trimmomatic (Bolger et al., 2014). Quality trimmed reads were assembled by Unicyler (Wick et al., 2017), and the presence of plasmids in the assembled contigs and sequencing reads was tested using the PlasmidFinder web server (Carattoli and Hasman, 2020). Identification of conjugative, mobilizable, or non-transferable elements among the possible plasmid contigs was performed using the OriT-Finder web server (Li et al., 2018). Contigs with plasmid-related elements were checked against the NCBI non-redundant nucleotide database using *blastn* program and filtered out from the assembly (Camacho et al., 2009). The remaining contigs were considered chromosomal DNA, and these contigs were reordered with Mauve using the genome of *S. epidermidis* ATCC 12228 (NC_004461) as a reference (Darling et al., 2004). The rearranged contigs were annotated with the stand-alone program Prokka (Seemann, 2014). Prophage sequences were identified using the Phaster web server (Arndt et al., 2016). Transposons were identified using *blastn* and the transposons listed in (Rauve et al., 2020). Screening for virulence factors was performed using the VFAnalyzer web server and the stand-alone *blastp* list of biofilm-associated proteins (coverage over 50%, identity over 30%) (**Table S11**) (Liu et al., 2019). Genomic determinants of antibiotic resistance were detected using the stand-alone *blastp* (coverage over 50%, identity over 30%) against a number of *Staphylococcus* resistance genes (**Table S12**). *In silico* screening of phage defense mechanisms was performed using the PADLOC web server (Payne et al., 2022).

## 3 Results

### 3.1 Phage isolation, morphology and host range

Initially, 20 *Staphylococcus-*specific phages were isolated from wastewater by enrichment with either *S. epidermidis* or *S. aureus* (**Table S1**). Most phages were isolated using COB-Sec1, COB-Sec2, COB-Sec4, COB-SeV2 (*S. epidermidis)*, COB-SA1 and COB-SA2 (*S. aureus*) as indicator strains. Twelve phages isolated from samples collected in winter were only *S. epidermidis*-specific (**Table S2**). Therefore, the next isolations were focused on isolating only phages that exhibited activity against both *S. epidermidis* and *S. aureus*, resulting in the isolation of 10 additional phages (**Table S2**).

**Figure 1:**
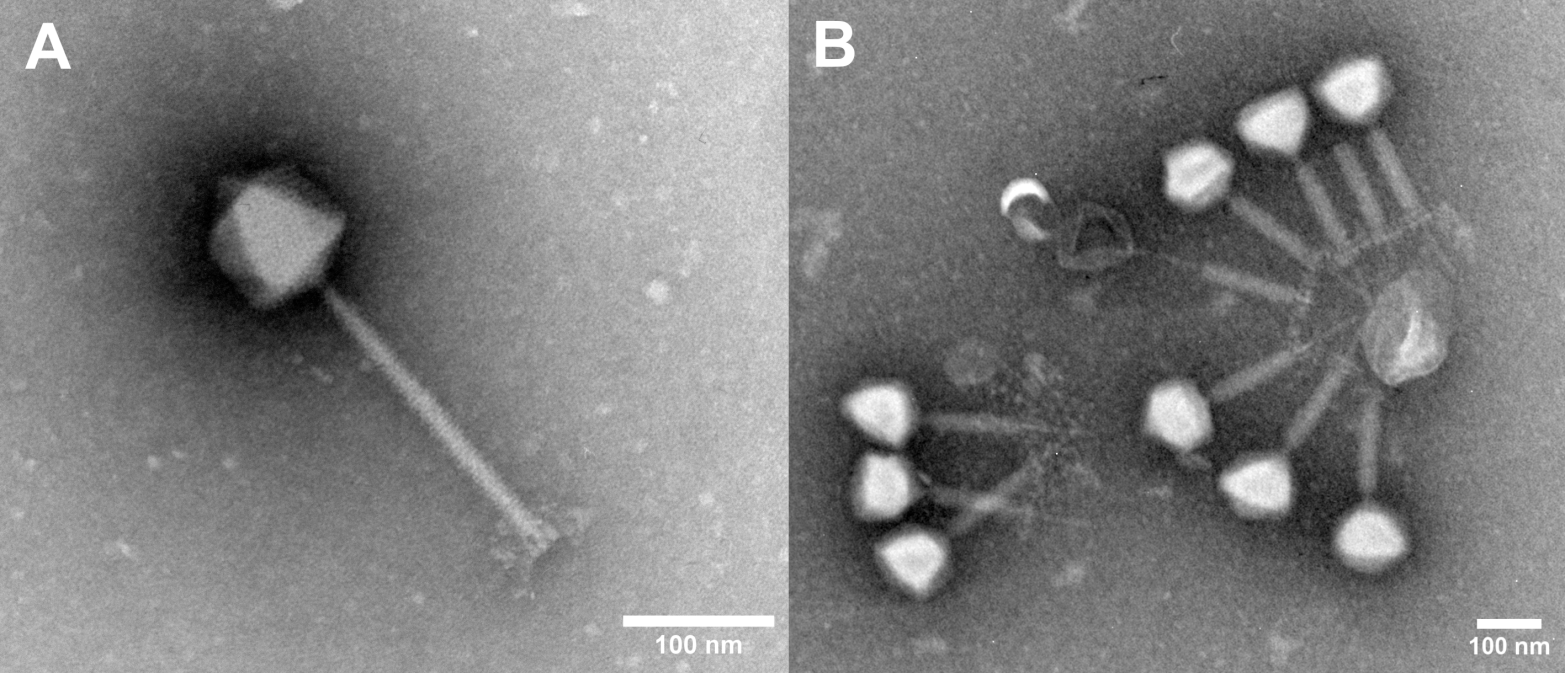
Electron micrographs of phage COP-80A **(A)** and phage aggregate of COP-E712 **(B)**. Scale bar = 100 nm.

The isolated phages have long contractile tails and icosahedral capsids that are typically described in the literature as myovirus or have siphovirus morphology (**Figure 1**; **Table S2**). Many phages were also found with contracted tails and/or bound around a spherical contaminant. Based on the literature screen, we decided to focus on phages with myovirus morphology, as these were recognized as virulent and effective against the target species. The host range of these selected phages was determined using a set of commensal, clinical, and veterinary strains of *S. epidermidis* (n=44), *S. aureus* (n=12), *S. pseudintermedius* (n=4), *S. pasteuri* (n=4), *S. lugdunensis* (n=2), *S. capitis* (n=1) and *S. pettenkoferi* (n=1) (**Figure S1**). Based on specificity, the isolated phages tested (n=13) could be divided into two groups. The first group, COP-80A, COP-80B, COP-110 and COP-E712, were highly *S. epidermidis-*specific. These phages infect also other coagulase-negative species (*S. pasteuri, S. lugdunensis*, and *S. capitis)*, but cannot infect strains of coagulase-positive species from the tested set of strains (*S. aureus* and *S. pseudintermedius*). The host ranges of COP-80A and COP-80B are very similar, but they exhibited different plaque morphologies. Additionally, the research phage bank of COP-80A was very unstable, its titer dropped for 1 log within one month during characterization, despite being propagated on the same strain and processed simultaneously with COP-80B.

The second group of phages lyses both species, but *S. aureus* to a greater extent. These phages also infect *S. pasteuri, S. lugdunensis* and *S. pettenkoferi* strains. None of the isolated phages were able to lyse tested *S. pseudintermedius* strains. A total of 10 of 44 *S. epidermidis* strains from our collection (23%) were not susceptible to any of the phages tested. COP-80A, COP-80B, and COP-110 infected the highest percentage of *S. epidermidis* strains tested, each infecting up to 68% of all strains.

### 3.2 Phage genome analysis

For each selected phage, *de novo* assemblies were created from quality trimmed reads and randomly subsampled reads. These were required to assemble a single contig with high coverage from all eight sequenced phages (**Table S3**). Phage lysates from COP-80A, COP-80B, and COB-110 (**Table S4**) were free of prophage contamination. However, contigs assembled from unmapped reads of phages propagated on the host *S. aureus* COB-SA1 (**Table S3**) were 18 kb or more in size. Considering possible contaminating prophage genomes, *blastn* was used against the Inphared database to identify the closest relatives and these were found among genera *Biseptimavirus* or *Siphoviridae* (**Table S4**). These phages were excluded from further analysis.

The closest relatives of the phages COP-80A, COP-80B, and COP-110 were phiIPLA-C1C and SEP1, both belonging to the genus *Sepunavirus* (ANI > 95%). Their genome size varied from 139 - 140 kb with 193 - 198 CDS and an average GC of 27.9%, comparable to other members of the *Sepunavirus* genus (**Table S5**). No tRNAs were identified in these genomes, and COB-110 carried three ncRNAs (group I catalytic intron, RAGATH RNA motif).

No similarities were found between the genomes of COP-80A, COP-80B, and COP-110 and the bacterial genomes available in the NCBI nonredundant nucleotide database. The PHACTS-predicted lytic values of these three phages were above 0.5 compared with the control *Staphylococcus* lysogenic phage with a lytic value of 0.33. No identifiable integrases or attachment sites were found in COP-80A, COP-80B, and COP-110, but homologs of putative transposases were detected (**Table S6**).

The genome termini were predicted by comparative analysis of the amino acid sequence of the large terminases. The large terminases of COP-80A, COP-80B, COP-110 were clustered together with *Listeria* A511 and other phages with direct repeat termini (DTR). More than 99.5% of the phage quality trimmed reads of COP-80A and COP-80B were mapped to the phage contig and less than 0.3% were assigned to the genome of the propagation host COB-Sec1 (**Table S8**). The phage reads covered the host genome unevenly, but the largest number of bases covered did not exceed 5 kb at low sequencing depth compared with the phage genome size (approximately 140 kb), which would be expected under generalized transduction. Because the propagation strain of COB-110 was not examined by sequencing, all three phages were propagated on the COB-Sec2 strain (see below) for further characterization.

None of the predicted phage genes had similarity to known virulence factors or antibiotic resistance associated genes.

### 3.3 Efficacy of phages against planktonic cultures and biofilms

**Figure 2:**
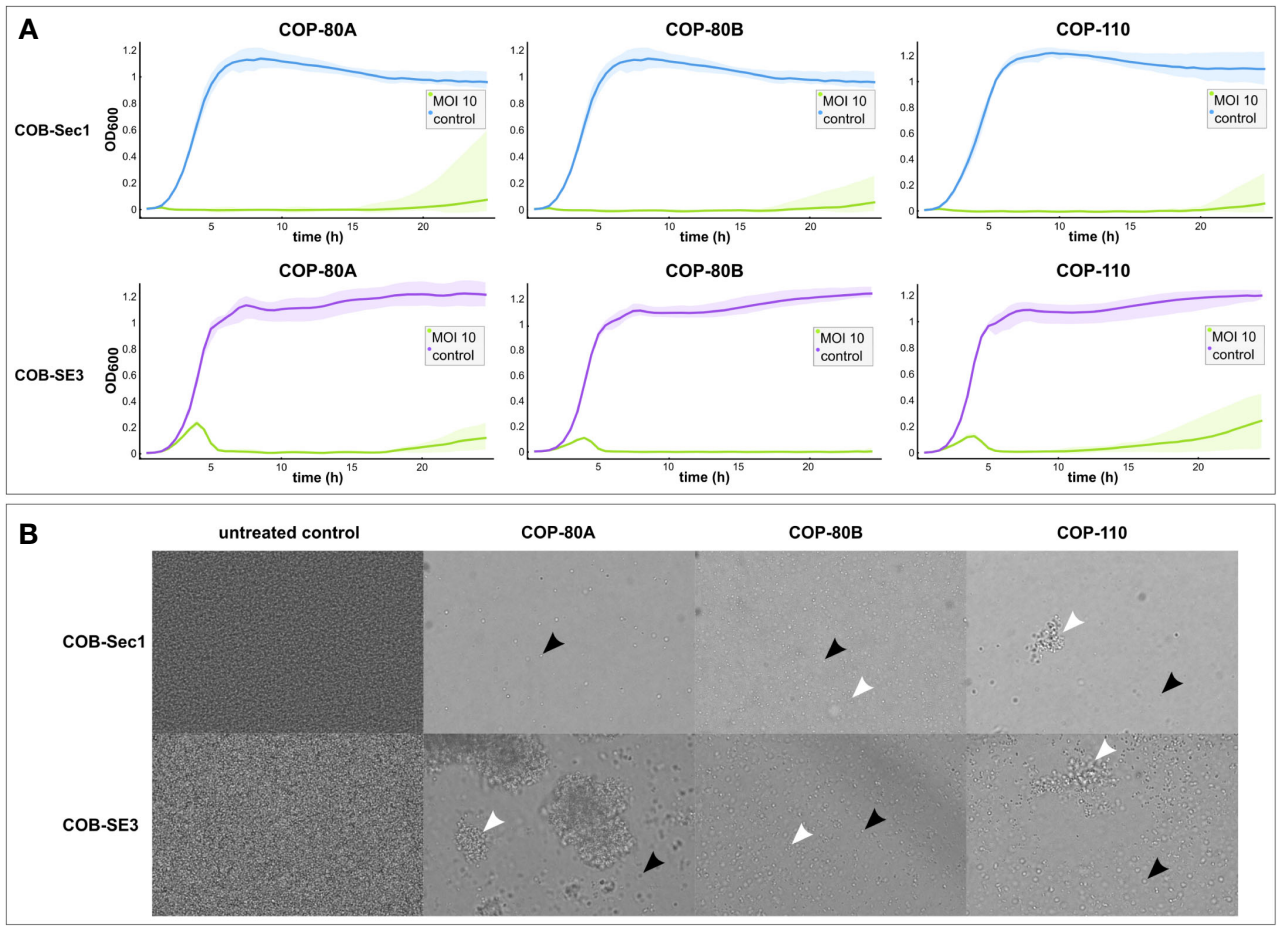
Antibacterial **(A)** and antibiofilm **(B)** activity of phages COP-80A, COP-80B and COP-110 against *S. epidermidis* COB-Sec1 (biofilm deficient) and COB-SE3 (biofilm proficient) at MOI 10. **(A)** Growth kinetics of COB-Sec1 and COB-SE3 in the presence of COP-80A, COP-80B and COP-110 was monitored for 24 h Results are shown as mean values of two experiments (biological replicates) with shaded area representing range of measured OD_600_. **(B)** Mature 24-hour biofilms of COB-Sec1 and COB-SE3 were treated with phages for 24 h and changes in their structure was determined by brightfield microscopy. Single cells are indicated by black arrows and cell aggregates by white arrows.

Efficacy of phages is linked to their antibacterial activity which was assessed by measuring growth kinetics (dynamic host range) of the biofilm-deficient commensal strain COB-Sec1 and biofilm-proficient clinical strain COB-SE3 growing in planktonic culture in the presence of isolated phages (**Table 1**; **Figure 2**). All three phages inhibited bacterial growth of the production host COB-Sec1 at MOI 10 for 20 h as well as clinical strain COB-SE3 (**Figure 2**), except the phage COP-110, which inhibited the growth of COB-SE3 for 16 h (**Table S15**; **Figure 2**). Duration of growth inhibition caused by the three tested phages compared with bacterial controls was significantly longer (p < 0.05) for COB-Sec1 at all MOIs tested. However, for COB-SE3 only higher MOIs of 1, 10 and 100 caused significantly longer (p < 0.05) growth inhibition compared to the control, where no inhibition was observed (**Table S15**). In general, COB-SE3 showed earlier re-growth than COB-Sec1, especially at MOI of 0.1 (**Figure S2**). The EOP analysis results are congruent with observations from the dynamic host range tests (effective lysis of COB-Sec1 and COB-SE3), and the decreased lysis efficiency of COB-SE8 was observed (**Table 1**).

Antibiofilm activity of isolated phages was evaluated by brightfield microscopy to obtain evidence on effect of phages on biofilm without disturbing its structure. The control biofilms of COB-Sec1 can be described as a layer of densely packed bacteria, while COB-SE3 biofilms also have denser areas where the shape of the bacteria could not be distinguished, possibly due to the presence of thick extracellular polysaccharides surrounding the microcolonies (**Figure 2**). Biofilms of COB-Sec1 challenged with selected phages were dispersed into individual floating bacteria while COB-SE3 biofilms disintegrated into aggregates of bacteria and possibly exopolysaccharides of different sizes (**Figure 2**). However, a notable decrease in bacterial numbers was observed in both strains. To cross-check results of host range and EOP tests, biofilms of COB-SE8 and three non-*S. epidermidis* strain biofilms were challenged with phages. All three phages dispersed biofilm of COB-SE8 causing similar effect as in COB-SE3 biofilm but to a lesser extent. Biofilm of *S. pasteuri* strain COB-SeS7 was efficiently disrupted by the three phages, while the biofilms of *S. lugdunensis* COB-SL1 and *S. capitis* COB-SC1 were not affected (**Figure S3**).

### 3.4 Characteristics of selected *S. epidermidis* hosts affecting phage efficacy

Four *S. epidermidis* strains with different clinical backgrounds and two commensal strains were compared with respect to their phage susceptibility (EOP), biofilm phenotype (crystal violet), and antibiotic resistance (antibiogram) as clinically relevant characteristics, and with respect to their genotype (**Table 1**). COB-Sec1 and COB-Sec2 are considered commensal strains because they originate from healthy human skin and were also the most susceptible strains to all isolated phages (**Figure S1**), serving as isolation strains and propagation hosts (**Table S2**). *De novo* assembly of the genomes of COB-Sec1 and COB-Sec2 resulted in multiple contigs, and those that showed similarities to existing plasmid sequences and plasmid elements were excluded, while the remaining contigs were considered to be chromosomal DNA (**Table S9**). The genome length of COB-Sec1 is 2.42 Mb and COB-Sec2 is 2.36 Mb with an average GC content of 32% (**Table S8**). Despite the presence of several transposase-related genes, no transposons were detected. Complete prophage sequences are not present in their genomes (**Table S10**). Homologs of virulence factors from commensal *S. epidermidis* ATCC 12228 are also present in COB-Sec1 and COB-Sec2, with the absence of IcaABCDR and toxins except for beta-hemolysin (**Table S11**). In contrast, COB-Sec1 and COB-Sec2 encode genes of cell wall proteins (Aap, Aae, Ses, Bap, Cid) associated with biofilm formation absent in ATCC 12228. Nevertheless, COB-Sec1 formed only a weak biofilm and COB-Sec2 formed a moderate biofilm (**Table 1**). Both strains were resistant to clindamycin but did not encode for its genetic determinant. COB-Sec1 carries more antibiotic resistance markers (BlaZ, NorA, FexA) compared to COB-Sec2 (**Table S12**). Among phage defense mechanisms, an incomplete restriction modification system (RMS) was identified in both strains. No repetitive CRISPR sequences were found. We considered COB-Sec1 and COB-Sec2 as suitable candidates for indicator and propagation strains because they pose a low risk of prophage induction or carryover of genes of concern.

COB-SE3 was found to be biofilm proficient and carries genes encoding IcaABCDR and cell wall proteins associated with an enhanced biofilm phenotype (**Tables 1**; **S11**). Its general genomic features were similar to those of COB-Sec1 and COB-Sec2, and it also lacks transposons and active prophage sequences. COB-SE3 was resistant to erythromycin and tetracycline, although corresponding resistance-associated genes were not detected. Propagation of isolated phages on COB-SE3 achieved the EOP of the propagating strain despite the ability to form a thick biofilm and the presence of RMS type II (*Sau*3AI) and DndFGH proteins, another restriction modification system associated with phosphorothioate modifications of host DNA (**Table S13**). Lower susceptibility to isolated phage was observed with COB-SE8 (**Table 1**). It formed moderate biofilm and carried several genetic determinants of antibiotic resistance, including MecA, BlaZ, Aac/Aph, NorA, and showed resistance to beta-lactams, aminoglycosides, and levofloxacin (**Table 1**). No active prophages were detected, so the reduced EOP may be due to the presence of the RMS type II (*Eco*RV), which is different from that present in the genome of COB-SE3 (**Table S13**). Two biofilm proficient strains, COB-SE6 and COB-SE11, were both resistant to isolated phages. They carry the same virulence factors, including IcaABCDR. COB-SE6 was resistant to multiple antibiotics, while COB-SE11 was resistant only to tetracycline. Although their general characteristics are similar to other sequenced *S. epidermidis* strains, a complete temperate phage genome is present in the genome of COB-SE11, whereas COB-SE6 carries two incomplete prophages and one that could also be complete. In addition to the possibly active complete temperate phage, the RMS-system II might affect phage propagation on the strain COB-SE11, while the abortive infection mechanism AbiK (48% identity and 99% coverage with *Lactococcus* AbiK (Q48614)) was found in the genome of COB-SE6.

## 4 Discussion

In the present work we have isolated several *Staphylococcus*-specific phages that are either specific for *S. epidermidis* or infect multiple species, but mostly *S. aureus*. Similar host range patterns of *Staphylococcus* specific phages have been observed previously but this requires further evidence as phages are typically tested against only a limited number of strains of non-*S. aureus* or non-*S. epidermidis* species. Wastewater has been shown to be a good source of multiple *Staphylococcus* phages, regardless of the collection season (Alves et al., 2014; Gutiérrez et al., 2015; Göller et al., 2021). Enrichment with a single strain of *S. epidermidis* selected for phages that had the morphology of siphoviruses although much larger diversity of phages was expected in the analyzed samples. By including *S. aureus* in the sample screening, we were able to select phages with myovirus morphology, which is consistent with the recent report on ecology of environmental staphylococcal phages (Göller et al., 2021). However, no phages with podovirus morphology were observed, although they had been previously isolated from wastewater samples (Cater et al., 2017). Additionally, isolated phages tend to cluster around unidentified structures, with their tails always directed toward the center of the aggregate as observed from electron micrographs. Many of them have contracted tails, possibly due to binding to a structure carrying a phage receptor resulting in a nonproductive DNA injection (Bae et al., 2006). The observed aggregates, if not disrupted prior to analysis, may lead to underestimate of phage titer by the established double agar overlay technique and other approaches to measure phage infectivity, which are important to phage production process analytics and studying phage numbers in *in vivo* experiments (Pantůček et al., 1998; Alves et al., 2014).

Selection of strains used to isolate phages is often neglected step in phage development that focuses on the number, diversity or potency of the isolated phages without considering the properties of the strain. Therefore, we decided to present phage isolation data using an uncharacterized but clinically relevant *S. aureus* strain causing prophage contamination in phage lysates to demonstrate the importance of performing basic phage and host characterization from the outset, e.g., prophage presence analysis. Since *S. aureus* COB-SA1 was not further analyzed, we hypothesize that a low induction of prophages occurred and that this could be detected due to the high sequencing coverage, which also led to fragmentation of the assembly in some phages, requiring more efforts in characterizing the genomes. Contamination of batches of lytic phages with temperate phages can be avoided by using staphylococcal strains lacking intact and inducible prophages. Although it is not known to what extent low-level temperate phages contamination may have affected treatment outcome in clinical phage therapy trials that did not present full sequencing results of the phage suspensions used, this type of contamination should be monitored and quantified when no prophage-free strains are available.

The three phages characterized in this study, COP-80A, COP-80B and COP-110, were free of prophage contamination. They belong to the genus *Sepunavirus* as they share more than > 95% ANI with the two exemplar isolates previously described as virulent phages with therapeutic potential against *S. epidermidis* (Melo et al., 2014; Gutiérrez et al., 2015). No evidence of lysogenicity was found, except for transposases that are commonly found in other *Sepunavirus* genomes but whose role has not yet been investigated (Gutiérrez et al., 2015). These phages also do not encode homologs of currently known virulence factors or genetic antibiotic resistance markers. Genome packaging could not be predicted from the sequencing data, but the large terminases of COP-80A, COP-80B, and COP-110 were clustered with *Listeria* phage A511, whose genome ends are experimentally proven to be direct terminal repeats (Klumpp et al., 2008). This genome structure suggests a packaging mechanism that is not associated with generalized transduction. From a genomic perspective, these phages carry a low risk of making the target bacteria more lethal. An important aspect in this regard is also the safety of the production organism being COB-Sec1 and COB-Sec2 in this study. *S. epidermidis* does not carry toxins commonly found in *S. aureus*, except for the gene encoding beta-hemolysin (Otto, 2012). This toxin is responsible for hydrolysis of sphingomyelin and is present in many other *S. epidermidis* strains, including O47, which does not exhibit hemolysis in the laboratory (Raue et al., 2020). Given the presence of beta-hemolysin, proteases, and lipases (**Table S11**) in the genomes of the production organism candidates COB-Sec1 and COB-Sec2, the activity of these enzymes should be measured for COB-Sec1 and COB-Sec2 to establish quality control measures for phage production for *in vivo* tests. The presence of genetic antibiotic resistance markers cannot be avoided in this species but can be minimized either by selection of strains with low resistance and careful monitoring of residual host DNA or by production on avirulent species, which would be possible given the host range of phages isolated from multiple species, but the efficiency of propagation would need to be improved (González-Menéndez et al., 2018).

Therapeutic potential of phages in the long-term largely depends on their activity against clinically relevant strains. *Staphylococcus* phages have already reached the level of clinical trials and are being used to treat infections in the context of expanded use, demonstrating their therapeutic potential and efforts to further develop this field (Lehman et al., 2019; Doub et al., 2020; Ferry et al., 2020; Van Nieuwenhuyse et al., 2021; Doub et al., 2022). Phages isolated in this study, COP-80A, COP-80B, and COP-110, infect the majority of *S. epidermidis* strains tested and also other coagulase-negative strains from our collection, similar as observed for phages phiIPLA-C1C and SEP1 (Melo et al., 2014; Gutiérrez et al., 2015). Beside the host range analysis, we evaluated their antibacterial activity by measuring bacterial growth kinetics in the presence of phages (dynamic host range) and examined their effect on biofilm morphology (antibiofilm activity). Bacterial growth monitoring can rely on optical density monitoring or cell respiration-based monitoring and is known as dynamic host range, which is often used as a rapid and informative method to estimate bacterial growth inhibition time by the phage and the emergence of a phage resistant subpopulation (Cooper et al., 2011; Henry et al., 2012). To demonstrate the differences in efficacy depending on the characteristics of the strain, the phages COP-80A, COP-80B, and COP-110 were tested on the production host COB-Sec1 and biofilm proficient clinical isolate COB-SE3. They were significantly more effective against the production organism for all MOIs tested, but the time they inhibited the growth of COB-SE3 was still longer than 15 h, which is similar to previous observations (Alves et al., 2014; Whittard et al., 2021). Since biofilm is critical for virulence of *S. epidermidis*, the effect on mature 24 h-old biofilm was examined using bright field microscopy. Biofilm proficient strains appear to be less susceptible to phage in biofilm, as previously observed, as they are dispersed into floating aggregates of various sizes, whereas weak biofilm producers are dispersed as individual floating bacteria (Melo et al., 2020). However, in both cases, disruption and a decrease in bacterial numbers was notable, as was the case with the *S. pasteuri* biofilm.

The observed biofilm structure disruption after phage treatment would need to be evaluated also by a quantitative approach at the next stage of phage antibiofilm activity characterization. Traditional quantitative methods include viability assay and staining of the biofilm (crystal violet, LIVE/DEAD staining, etc.) which provide complementary information on changes in biofilm thus supporting the understanding of phage efficacy. The crystal violet, the golden standard for biofilm analysis, showed large variations during the characterization of the biofilm phenotype in this study, and we did not find it informative enough to estimate the effect of the phages (Latka and Drulis-Kawa, 2020). Thus, the viability assay would be more informative, but based on the observed changes in biofilm structure in this study, extensive optimization is expected to ensure complete disaggregation of biofilm bacteria where measurements of viable bacteria would result from individual cells and not aggregates, preventing potential erroneous results on antibiofilm activity of phages.

The biofilm forming ability of the sequenced strains was consistent with the presence of IcaABDR, which is responsible for intercellular adhesion through intercellular polysaccharide adhesin (PIA) and is critical for biofilm formation (Cramton et al., 1999). However, *S. epidermidis* is also capable of transitioning to proteinaceous biofilms independent of PIA synthesis, but cell wall proteins known to be involved in this process did not contribute to the observed phenotype, which may also be due to the biofilm screening method chosen (Hennig et al., 2007). Biofilm forming ability did not limit the efficacy of the isolated phages when tested on COB-SE3, so the reason for resistance to the phages in COB-SE8, COB-SE6, and COB-SE11 may be the absence of a receptor or presence of other resistance mechanism. The lower EOP on COB-SE8 could be a result of RMS activity with *Eco*RV-like restriction enzyme, since its homologs are found only in a limited number of *S. epidermidis* genomes. The most common RMS type II system in *S. epidermidis* is *Sau*3A, which is also present in COB-SE3 and to which phages have become resistant, including isolated phages. The resistance of COB-SE11 could also result from superinfection immunity caused by a complete prophage in its genome. This mechanism has not yet been studied in *Staphylococcus*, but prophages are common in their genomes. A less common resistance mechanism, the abortive infection (AbiK), which has been described in *Lactococcus* as a protein that prevents maturation of the phage head, has been discovered in COB-SE6, but its relevance in resistance to lytic phages in *Staphylococcus* needs further investigation (Boucher et al., 2000).

## 5 Conclusion

We report the isolation of three novel virulent *Sepunavirus* phages, COP-80A, COP-80B and COP-110, capable of infecting a variety of *S. epidermidis* strains. Their activity has been demonstrated against planktonic bacteria and also against bacterial biofilms. No evidence for lysogenic lifestyle, generalized transduction or presence of undesirable genetic elements was found by genomic analysis suggesting they are suitable candidates for further safety and efficacy testing, either alone or in the form of a phage cocktail to achieve broad multispecies spectrum. We also propose bacterial resistance mechanisms to the phages studied. The article also highlights often overlooked aspects of phage preparation that make important contributions to the safety and success of phage therapy.

## Data availability statement

Bacterial genome sequences were deposited in the GenBank repository and are available under accession numbers JARGDX000000000 (COB-Sec1), JARGDW000000000 (COB-Sec2), JARGEB000000000 (COB-SE3), JARGEA000000000 (COB-SE6), JARGDZ000000000 (COB-SE8), JARGDY000000000 (COB-SE11). Phage genome sequences are available under accession numbers OQ448193 (COP-80A), OQ448194 (COP-80B), and OQ448195 (COP-110).

## Author contributions

VŠ: methodology, investigation, writing original draft, writing – review & editing. MM, JT, NP, MK, KŠ: investigation. SH: writing – review & editing. NJ: conceptualization, methodology, investigation, writing original draft, writing – review & editing. MP: funding acquisition, conceptualization, methodology, writing – review & editing.

## Footnotes

1
https://github.com/lh3/seqtk

2
http://enve-omics.ce.gatech.edu/ani/

3
https://blast.ncbi.nlm.nih.gov/Blast.cgi

## References

[B1] Alves D. R. Gaudion A. Bean J. E. Perez Esteban P. Arnot T. C. Harper D. R.. (2014). Combined use of bacteriophage K and a novel bacteriophage to reduce staphylococcus aureus biofilm formation. Appl. Environ. Microbiol. 80, 6694–6703. doi: 10.1128/AEM.01789-14 25149517 PMC4249044

[B2] Arndt D. Grant J. R. Marcu A. Sajed T. Pon A. Liang Y.. (2016). PHASTER: a better, faster version of the PHAST phage search tool. Nucleic Acids Res. 44, W16–W21. doi: 10.1093/NAR/GKW387 27141966 PMC4987931

[B3] Bae T. Baba T. Hiramatsu K. Schneewind O. (2006). Prophages of staphylococcus aureus Newman and their contribution to virulence. Mol. Microbiol. 62, 1035–1047. doi: 10.1111/j.1365-2958.2006.05441.x 17078814

[B4] Bolger A. M. Lohse M. Usadel B. (2014). Trimmomatic: a flexible trimmer for illumina sequence data. Bioinformatics 30, 2114–2120. doi: 10.1093/bioinformatics/btu170 24695404 PMC4103590

[B5] Bonilla N. Rojas M. I. Cruz G. N. F. Hung S. H. Rohwer F. Barr J. J. (2016). Phage on tap-a quick and efficient protocol for the preparation of bacteriophage laboratory stocks. PeerJ 4, e2261. doi: 10.7717/peerj.2261 27547567 PMC4975003

[B6] Boucher I. Émond E. Dion E. Montpetit D. Moineau S. (2000). Microbiological and molecular impacts of AkiK on the lytic cycle of lactococcus lactis phages of the 936 and P335 species. Microbiology 146, 445–453. doi: 10.1099/00221287-146-2-445 10708383

[B7] Camacho C. Coulouris G. Avagyan V. Ma N. Papadopoulos J. Bealer K.. (2009). BLAST+: architecture and applications. BMC Bioinf. 10, 1–9. doi: 10.1186/1471-2105-10-421 PMC2803857 20003500

[B8] Carattoli A. Hasman H. (2020). “PlasmidFinder and in silico pMLST: identification and typing of plasmid replicons in whole-genome sequencing (WGS),” in Horizontal gene transfer: methods and protocols. Ed. de la Cruz F. (New York, NY: Humana), 285–294. doi: 10.1007/978-1-4939-9877-7_20 31584170

[B9] Carlson K. (2005). “Appendix: working with bacteriophages,” in Bacteriophages: biology and applications. Eds. Kutter E. Sulakvelidze A. (Boca Raton, FL: CRC Press), 437–494.

[B10] Cater K. Dandu V. S. Bari S. M. N. Lackey K. Everett G. F. K. Hatoum-Aslan A. (2017). A novel staphylococcus podophage encodes a unique lysin with unusual modular design. mSphere 2, e00040–17. doi: 10.1128/mSphere.00040-17 28357414 PMC5362749

[B11] Cerca N. Oliveira R. Azeredo J. (2007). Susceptibility of staphylococcus epidermidis planktonic cells and biofilms to the lytic action of staphylococcus bacteriophage K. Lett. Appl. Microbiol. 45, 313–317. doi: 10.1111/J.1472-765X.2007.02190.X 17718845

[B12] Cook R. Brown N. Redgwell T. Rihtman B. Barnes M. Clokie M.. (2021). INfrastructure for a PHAge REference database: identification of Large-scale biases in the current collection of cultured phage genomes. PHAGE 2, 214–223. doi: 10.1089/phage.2021.0007 36159887 PMC9041510

[B13] Cooper C. J. Denyer S. P. Maillard J. Y. (2011). Rapid and quantitative automated measurement of bacteriophage activity against cystic fibrosis isolates of pseudomonas aeruginosa. J. Appl. Microbiol. 110, 631–640. doi: 10.1111/J.1365-2672.2010.04928.X 21205097

[B14] Cramton S. E. Gerke C. Schnell N. F. Nichols W. W. Götz F. (1999). The intercellular adhesion (ica) locus is present in staphylococcus aureus and is required for biofilm formation. Infect. Immun. 67, 5427–5433. doi: 10.1128/IAI.67.10.5427-5433.1999 10496925 PMC96900

[B15] Danecek P. Bonfield J. K. Liddle J. Marshall J. Ohan V. Pollard M. O.. (2021). Twelve years of SAMtools and BCFtools. Gigascience 10, 1–4. doi: 10.1093/gigascience/giab008 PMC7931819 33590861

[B16] Darling A. C. E. Mau B. Blattner F. R. Perna N. T. (2004). Mauve: multiple alignment of conserved genomic sequence with rearrangements. Genome Res. 14, 1394–1403. doi: 10.1101/GR.2289704 15231754 PMC442156

[B17] Darouiche R. O. (2004). Treatment of infections associated with surgical implants. N. Engl. J. Med. 350, 1422–1429. doi: 10.1056/NEJMra035415 15070792

[B18] Demšar J. Curk T. Erjavec A. Gorup Č. Hočevar T. Milutinovič M.. (2013). Orange: data mining toolbox in Python. J. Mach. Learn. Res. 14, 2349–2353.

[B19] Doub J. B. Ng V. Y. Johnson A. J. Slomka M. Fackler J. Horne B.. (2020). Salvage bacteriophage therapy for a chronic MRSA prosthetic joint infection. Antibiotics 9, 241. doi: 10.3390/antibiotics9050241 32397354 PMC7277870

[B20] Doub J. B. Ng V. Y. Lee M. Chi A. Lee A. Würstle S.. (2022). Salphage: salvage bacteriophage therapy for recalcitrant MRSA prosthetic joint infection. Antibiotics 11, 616. doi: 10.3390/antibiotics11050616 35625260 PMC9137795

[B21] Doub J. B. Ng V. Y. Wilson E. Corsini L. Chan B. K. (2021). Successful treatment of a recalcitrant staphylococcus epidermidis prosthetic knee infection with intraoperative bacteriophage therapy. Pharmaceuticals 14, 231. doi: 10.3390/ph14030231 33800146 PMC7998749

[B22] Fanaei Pirlar R. Wagemans J. Ponce Benavente L. Lavigne R. Trampuz A. Gonzalez Moreno M. (2022). Novel bacteriophage specific against staphylococcus epidermidis and with antibiofilm activity. Viruses 14, 1340. doi: 10.3390/v14061340 35746811 PMC9230115

[B23] FDA, Center for Biologics evaluation and research, and National Institute of Allergy and Infectious Disease (2021) Science and regulation of bacteriophage therapy (Washington, D. C). Available at: https://www.fda.gov/media/159399/download (Accessed April 22, 2023).

[B24] Fernández L. González S. Campelo A. B. Martínez B. Rodríguez A. García P. (2017). Low-level predation by lytic phage phiIPLA-RODI promotes biofilm formation and triggers the stringent response in staphylococcus aureus. Sci. Rep. 7, 40965. doi: 10.1038/srep40965 28102347 PMC5244418

[B25] Ferry T. Kolenda C. Batailler C. Gustave C. A. Lustig S. Malatray M.. (2020). Phage therapy as adjuvant to conservative surgery and antibiotics to salvage patients with relapsing s. aureus prosthetic knee infection. Front. Med. 7. doi: 10.3389/fmed.2020.570572 PMC7701306 33304911

[B26] Fish R. Kutter E. Bryan D. Wheat G. Kuhl S. (2018). Resolving digital staphylococcal osteomyelitis using bacteriophage–a case report. Antibiotics 7, 87. doi: 10.3390/antibiotics7040087 30279396 PMC6316425

[B27] Göller P. C. Elsener T. Lorgé D. Radulovic N. Bernardi V. Naumann A.. (2021). Multi-species host range of staphylococcal phages isolated from wastewater. Nat. Commun. 12, 1–17. doi: 10.1038/s41467-021-27037-6 34845206 PMC8629997

[B28] González-Menéndez E. Arroyo-López F. N. Martínez B. García P. Garrido-Fernández A. Rodríguez A. (2018). Optimizing propagation of staphylococcus aureus infecting bacteriophage vB_SauM-phiIPLA-RODI on staphylococcus xylosus using response surface methodology. Viruses 10, 153. doi: 10.3390/V10040153 29584701 PMC5923447

[B29] Gutiérrez D. Vandenheuvel D. Martínez B. Rodríguez A. Lavigne R. García P. (2015). Two phages, phiIPLA-RODI and phiIPLA-C1C, lyse mono-and dual-species staphylococcal biofilms. Appl. Environ. Microbiol. 81, 3336–3348. doi: 10.1128/AEM.03560-14 25746992 PMC4407228

[B30] Hennig S. Nyunt Wai S. Ziebuhr W. (2007). Spontaneous switch to PIA-independent biofilm formation in an ica-positive staphylococcus epidermidis isolate. Int. J. Med. Microbiol. 297, 117–122. doi: 10.1016/J.IJMM.2006.12.001 17292669

[B31] Henry M. Biswas B. Vincent L. Mokashi V. Schuch R. Bishop-Lilly K. A.. (2012). Development of a high throughput assay for indirectly measuring phage growth using the OmniLogTM system. Bacteriophage 2, 159–167. doi: 10.4161/BACT.21440 23275867 PMC3530525

[B32] Jurado A. Fernández L. Rodríguez A. García P. (2022). Understanding the mechanisms that drive phage resistance in staphylococci to prevent phage therapy failure. Viruses 14, 1061. doi: 10.3390/V14051061 35632803 PMC9146914

[B33] Kandel C. E. Jenkinson R. Daneman N. Backstein D. Hansen B. E. Muller M. P.. (2019). Predictors of treatment failure for hip and knee prosthetic joint infections in the setting of 1-and 2-stage exchange arthroplasty: a multicenter retrospective cohort. Open Forum Infect. Dis. 6, ofz452. doi: 10.1093/ofid/ofz452 31737739 PMC6847009

[B34] Kifelew L. G. Warner M. S. Morales S. Vaughan L. Woodman R. Fitridge R.. (2020). Efficacy of phage cocktail AB-SA01 therapy in diabetic mouse wound infections caused by multidrug-resistant staphylococcus aureus. BMC Microbiol. 20, 1–10. doi: 10.1186/S12866-020-01891-8 32646376 PMC7346408

[B35] Klumpp J. Dorscht J. Lurz R. Bielmann R. Wieland M. Zimmer M.. (2008). The terminally redundant, nonpermuted genome of listeria bacteriophage A511: a model for the SPO1-like myoviruses of gram-positive bacteria. J. Bacteriol. 190, 5753–5765. doi: 10.1128/JB.00461-08 18567664 PMC2519532

[B36] Kropinski A. M. Mazzocco A. Waddell T. E. Lingohr E. Johnson R. P. (2009). “Enumeration of bacteriophages by double agar overlay plaque assay,” in Bacteriophages: methods and protocols, volume 1: isolation, characterization, and interactions. Eds. Clokie M. R. J. Kropinski A. M. (Totowa, NJ: Humana Press), 69–76. doi: 10.1007/978-1-60327-164-6_7 19066811

[B37] Kutter E. (2009). “Phage host range and efficiency of plating,” in Bacteriophages: methods and protocols, volume 1: isolation, characterization, and interactions. Eds. Clokie M. R. J. Kropinski A. M. (Totowa, NJ: Humana Press), 141–149. doi: 10.1007/978-1-60327-164-6_14 19066818

[B38] Kvachadze L. Balarjishvili N. Meskhi T. Tevdoradze E. Skhirtladze N. Pataridze T.. (2011). Evaluation of lytic activity of staphylococcal bacteriophage Sb-1 against freshly isolated clinical pathogens. Microb. Biotechnol. 4, 643–650. doi: 10.1111/J.1751-7915.2011.00259.X 21481199 PMC3819013

[B39] Kwasny S. M. Opperman T. J. (2010). Static biofilm cultures of gram-positive pathogens grown in a microtiter format used for anti-biofilm drug discovery. Curr. Protoc. Pharmacol. 50, 13A.8.1–13A.8.23. doi: 10.1002/0471141755.ph13a08s50 PMC3272335 22294365

[B40] Latka A. Drulis-Kawa Z. (2020). Advantages and limitations of microtiter biofilm assays in the model of antibiofilm activity of klebsiella phage KP34 and its depolymerase. Sci. Rep. 10, 20338. doi: 10.1038/s41598-020-77198-5 33230270 PMC7683578

[B41] Lehman S. M. Mearns G. Rankin D. Cole R. A. Smrekar F. Branston S. D.. (2019). Design and preclinical development of a phage product for the treatment of antibiotic-resistant staphylococcus aureus infections. Viruses 11, 88. doi: 10.3390/v11010088 30669652 PMC6356596

[B42] Leskinen K. Tuomala H. Wicklund A. Horsma-Heikkinen J. Kuusela P. Skurnik M.. (2017). Characterization of vB_SauM-fRuSau02, a twort-like bacteriophage isolated from a therapeutic phage cocktail. Viruses 9, 258. doi: 10.3390/V9090258 28906479 PMC5618024

[B43] Li H. (2013). Aligning sequence reads, clone sequences and assembly contigs with BWA-MEM. arXiv. doi: 10.48550/arXiv.1303.3997

[B44] Li X. Xie Y. Liu M. Tai C. Sun J. Deng Z.. (2018). oriTfinder: a web-based tool for the identification of origin of transfers in DNA sequences of bacterial mobile genetic elements. Nucleic Acids Res. 46, W229–W234. doi: 10.1093/NAR/GKY352 29733379 PMC6030822

[B45] Liu B. Zheng D. Jin Q. Chen L. Yang J. (2019). VFDB 2019: a comparative pathogenomic platform with an interactive web interface. Nucleic Acids Res. 47, D687–D692. doi: 10.1093/NAR/GKY1080 30395255 PMC6324032

[B46] Loc-Carrillo C. Abedon S. T. (2011). Pros and cons of phage therapy. Bacteriophage 1, 111. doi: 10.4161/BACT.1.2.14590 22334867 PMC3278648

[B47] Mangalea M. R. Duerkop B. A. (2020). Fitness trade-offs resulting from bacteriophage resistance potentiate synergistic antibacterial strategies. Infect. Immun. 88, e00926–19. doi: 10.1128/IAI.00926-19 32094257 PMC7309606

[B48] McArthur A. G. Waglechner N. Nizam F. Yan A. Azad M. A. Baylay A. J.. (2013). The comprehensive antibiotic resistance database. Antimicrob. Agents Chemother. 57, 3348–3357. doi: 10.1128/AAC.00419-13 23650175 PMC3697360

[B49] McNair K. Bailey B. A. Edwards R. A. (2012). PHACTS, a computational approach to classifying the lifestyle of phages. Bioinformatics 28, 614–618. doi: 10.1093/BIOINFORMATICS/BTS014 22238260 PMC3289917

[B50] Melo L. D. R. França A. Brandão A. Sillankorva S. Cerca N. Azeredo J. (2018). Assessment of Sep1virus interaction with stationary cultures by transcriptional and flow cytometry studies. FEMS Microbiol. Ecol. 94, 143. doi: 10.1093/FEMSEC/FIY143 30060135

[B51] Melo L. D. R. Pinto G. Oliveira F. Vilas-Boas D. Almeida C. Sillankorva S.. (2020). The protective effect of staphylococcus epidermidis biofilm matrix against phage predation. Viruses 12, 1076. doi: 10.3390/V12101076 32992766 PMC7601396

[B52] Melo L. D. R. Sillankorva S. Ackermann H. W. Kropinski A. M. Azeredo J. Cerca N. (2014). Isolation and characterization of a new staphylococcus epidermidis broad-spectrum bacteriophage. J. Gen. Virol. 95, 506–515. doi: 10.1099/vir.0.060590-0 24189619

[B53] Merabishvili M. Pirnay J. P. Verbeken G. Chanishvili N. Tediashvili M. Lashkhi N.. (2009). Quality-controlled small-scale production of a well-defined bacteriophage cocktail for use in human clinical trials. PloS One 4, e4944. doi: 10.1371/JOURNAL.PONE.0004944 19300511 PMC2654153

[B54] Merrill B. D. Ward A. T. Grose J. H. Hope S. (2016). Software-based analysis of bacteriophage genomes, physical ends, and packaging strategies. BMC Genomics 17, 1–16. doi: 10.1186/s12864-016-3018-2 27561606 PMC5000459

[B55] Moller A. G. Lindsay J. A. Read T. D. (2019). Determinants of phage host range in staphylococcus species. Appl. Environ. Microbiol. 85, e00209–19. doi: 10.1128/AEM.00209-19 30902858 PMC6532042

[B56] O’Flaherty S. Ross R. P. Meaney W. Fitzgerald G. F. Elbreki M. F. Coffey A. (2005). Potential of the polyvalent anti-staphylococcus bacteriophage K for control of antibiotic-resistant staphylococci from hospitals. Appl. Environ. Microbiol. 71, 1836. doi: 10.1128/AEM.71.4.1836-1842.2005 15812009 PMC1082512

[B57] Olsen G. J. Lane D. J. Giovannoni S. J. Pace N. R. Stahl D. A. (1986). Microbial ecology and evolution: a ribosomal RNA approach. Annu. Rev. Microbiol. 40, 337–365. doi: 10.1146/annurev.mi.40.100186.002005 2430518

[B58] Onsea J. Soentjens P. Djebara S. Merabishvili M. Depypere M. Spriet I.. (2019). Bacteriophage application for difficult-To-Treat musculoskeletal infections: development of a standardized multidisciplinary treatment protocol. Viruses 11, 891. doi: 10.3390/V11100891 31548497 PMC6832313

[B59] Otto M. (2009). Staphylococcus epidermidis - the “accidental” pathogen. Nat. Rev. Microbiol. 7, 555–567. doi: 10.1038/nrmicro2182 19609257 PMC2807625

[B60] Otto M. (2012). Molecular basis of staphylococcus epidermidis infections. Semin. Immunopathol. 34, 214. doi: 10.1007/S00281-011-0296-2 PMC3272124 22095240

[B61] Pantůček R. Rosypalová A. Doškař J. Kailerová J. Růžičková V. Borecká P.. (1998). The polyvalent staphylococcal phage φ812: its host-range mutants and related phages. Virology 246, 241–252. doi: 10.1006/VIRO.1998.9203 9657943

[B62] Patel R. (2023). Periprosthetic joint infection. N. Engl. J. Med. 388, 251–262. doi: 10.1056/NEJMRA2203477 36652356

[B63] Payne L. J. Meaden S. Mestre M. R. Palmer C. Toro N. Fineran P. C.. (2022). PADLOC: a web server for the identification of antiviral defence systems in microbial genomes. Nucleic Acids Res. 50, W541–W550. doi: 10.1093/NAR/GKAC400 35639517 PMC9252829

[B64] Pelfrene E. Willebrand E. Cavaleiro Sanches A. Sebris Z. Cavaleri M. (2016). Bacteriophage therapy: a regulatory perspective. J. Antimicrob. Chemother. 71, 2071–2074. doi: 10.1093/JAC/DKW083 27068400

[B65] Pirnay J. P. Blasdel B. G. Bretaudeau L. Buckling A. Chanishvili N. Clark J. R.. (2015). Quality and safety requirements for sustainable phage therapy products. Pharm. Res. 32, 2173–2179. doi: 10.1007/S11095-014-1617-7 25585954 PMC4452253

[B66] Plaut R. D. Stibitz S. (2019). “Regulatory considerations for bacteriophage therapy products,” in Phage Therapy: A Practical Approach (Springer International Publishing), 337–349. doi: 10.1007/978-3-030-26736-0_13

[B67] Prjibelski A. Antipov D. Meleshko D. Lapidus A. Korobeynikov A. (2020). Using SPAdes *De novo* assembler. Curr. Protoc. Bioinforma 70, e102. doi: 10.1002/CPBI.102 32559359

[B68] Raue S. Fan S. H. Rosenstein R. Zabel S. Luqman A. Nieselt K.. (2020). The genome of staphylococcus epidermidis O47. Front. Microbiol. 11, 2061. doi: 10.3389/fmicb.2020.02061 32983045 PMC7477909

[B69] Rupp M. E. Archer G. L. (1994). Coagulase-negative staphylococci: pathogens associated with medical progress. Clin. Infect. Dis. 19, 231–243. doi: 10.1093/clinids/19.2.231 7986894

[B70] Schneider C. A. Rasband W. S. Eliceiri K. W. (2012). NIH Image to ImageJ: 25 years of image analysis. Nat. Methods 9, 671–675. doi: 10.1038/nmeth.2089 22930834 PMC5554542

[B71] Seemann T. (2014). Prokka: rapid prokaryotic genome annotation. Bioinformatics 30, 2068–2069. doi: 10.1093/BIOINFORMATICS/BTU153 24642063

[B72] Shen A. Millard A. (2021). Phage genome annotation: where to begin and end. PHAGE Ther. Appl. Res. 2, 183–193. doi: 10.1089/phage.2021.0015 PMC9041514 36159890

[B73] Shimamori Y. Pramono A. K. Kitao T. Suzuki T. Aizawa S. Kubori T.. (2021). Isolation and characterization of a novel phage SaGU1 that infects staphylococcus aureus clinical isolates from patients with atopic dermatitis. Curr. Microbiol. 78, 1267–1276. doi: 10.1007/s00284-021-02395-y 33638001 PMC7997843

[B74] Terzian P. Olo Ndela E. Galiez C. Lossouarn J. Pérez Bucio R. E. Mom R.. (2021). PHROG: families of prokaryotic virus proteins clustered using remote homology. NAR Genomics Bioinforma 3, lqab067. doi: 10.1093/NARGAB/LQAB067 PMC8341000 34377978

[B75] Turner D. Adriaenssens E. M. Tolstoy I. Kropinski A. M. (2021). Phage annotation guide: guidelines for assembly and high-quality annotation. PHAGE Ther. Appl. Res. 2, 170–182. doi: 10.1089/phage.2021.0013 PMC8785237 35083439

[B76] Valente L. G. Pitton M. Fürholz M. Oberhaensli S. Bruggmann R. Leib S. L.. (2021). Isolation and characterization of bacteriophages from the human skin microbiome that infect staphylococcus epidermidis. FEMS Microbes 2, xtab003. doi: 10.1093/femsmc/xtab003 PMC10117716 37334235

[B79] Vandersteegen K. Kropinski A. M. Nash J. H. E. Noben J.-P. Hermans K. Lavigne R. (2013). Romulus And remus, two phage isolates representing a distinct clade within the twortlikevirus genus, display suitable properties for phage therapy applications. J. Virol. 87, 3237–3247. doi: 10.1128/jvi.02763-12 23302893 PMC3592175

[B77] Van Epps J. S. Younger J. G. (2016). Implantable device-related infection. Shock 46, 597–608. doi: 10.1097/SHK.0000000000000692 27454373 PMC5110396

[B78] Van Nieuwenhuyse B. Galant C. Brichard B. Docquier P. L. Djebara S. Pirnay J. P.. (2021). A case of *in situ* phage therapy against staphylococcus aureus in a bone allograft polymicrobial biofilm infection: outcomes and phage-antibiotic interactions. Viruses 13, 1898. doi: 10.3390/v13101898 34696328 PMC8539586

[B80] Weisburg W. G. Barns S. M. Pelletier D. A. Lane D. J. (1991). 16S ribosomal DNA amplification for phylogenetic study. J. Bacteriol 173, 697–703. doi: 10.1128/JB.173.2.697-703.1991 1987160 PMC207061

[B81] Whittard E. Redfern J. Xia G. Millard A. Ragupathy R. Malic S.. (2021). Phenotypic and genotypic characterization of novel polyvalent bacteriophages with potent *In vitro* activity against an international collection of genetically diverse staphylococcus aureus. Front. Cell. Infect. Microbiol. 11. doi: 10.3389/fcimb.2021.698909 PMC8290860 34295840

[B82] Wick R. R. Judd L. M. Gorrie C. L. Holt K. E. (2017). Unicycler: resolving bacterial genome assemblies from short and long sequencing reads. PloS Comput. Biol. 13, e1005595. doi: 10.1371/JOURNAL.PCBI.1005595 28594827 PMC5481147

[B83] Wisplinghoff H. Bischoff T. Tallent S. M. Seifert H. Wenzel R. P. Edmond M. B. (2004). Nosocomial bloodstream infections in US hospitals: analysis of 24,179 cases from a prospective nationwide surveillance study. Clin. Infect. Dis. 309, 309–317. doi: 10.1086/421946 15306996

[B84] Zdobnov E. M. Apweiler R. (2001). InterProScan – an integration platform for the signature-recognition methods in InterPro. Bioinformatics 17, 847–848. doi: 10.1093/BIOINFORMATICS/17.9.847 11590104

[B85] Zimmerli W. (2014). Clinical presentation and treatment of orthopaedic implant-associated infection. J. Intern. Med. 276, 111–119. doi: 10.1111/JOIM.12233 24605880

