# Supplementary material for: Isolation and in vitro characterization of novel S. epidermidis phages for therapeutic applications
Source: Front Cell Infect Microbiol. 2023 May 24;13:1169135. doi: 10.3389/fcimb.2023.1169135 (PMC10244729; doi:10.3389/fcimb.2023.1169135)
Supplement: Supplementary file 1 [file DataSheet_1.docx]

Supplementary Material

# Supplementary Data

# Supplementary Figures and Tables

## Supplementary Figures


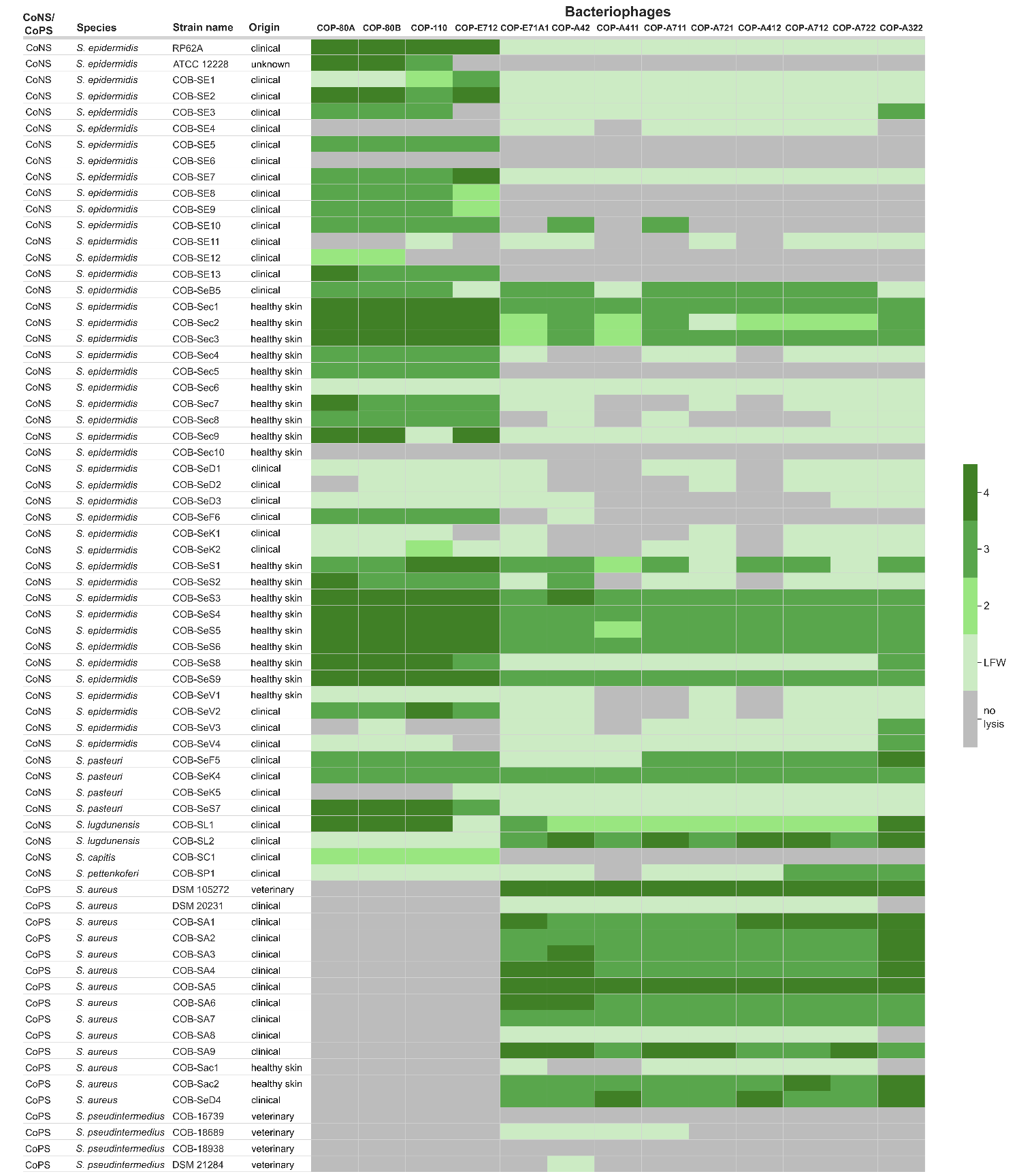


**Supplementary Figure 1.** **Host range of the isolated phages.**

Clinical strains were obtained from Valdoltra Orthopaedic Hospital and were isolated from patients with orthopedic-related infections. Strains RP62A (DSM 28319), DSM 20231, DSM 105272, and DSM 21284 were purchased from DSMZ - German Collection of Microorganisms and Cell Cultures (Germany) and strain ATCC 12228 (NCTC 13360) from National Collection of Type Cultures (UK). Phages COP-80A, COP-80B, COP-110 and COP-E712 were propagated and titrated on *S. epidermidis* strain COB-Sec2, whereas phages COP-E71A1, COP-A42, COP-A411, COP-A711, COP-A721, COP-A412, COP-A712, COP-A722 and COP-A322 were propagated and titrated on *S. aureus* strain COB-SA1. Spots on bacterial lawns were examined visually, graded as shown in the legend, and depicted as a heat map where each grade is color-coded: complete clearing is depicted as the darkest green color (grade 4), slightly turbid lysis zone as slightly lighter green (grade 3), a few individual plaques as lime green color (grade 2), and no clearing is visualized as grey (grade 0). When a lysis halo was observed on the bacterial lawn this was graded as ‘lysis from without’ and the strain was considered not infected, this is visualized as the lightest green color. LFW – lysis from without.


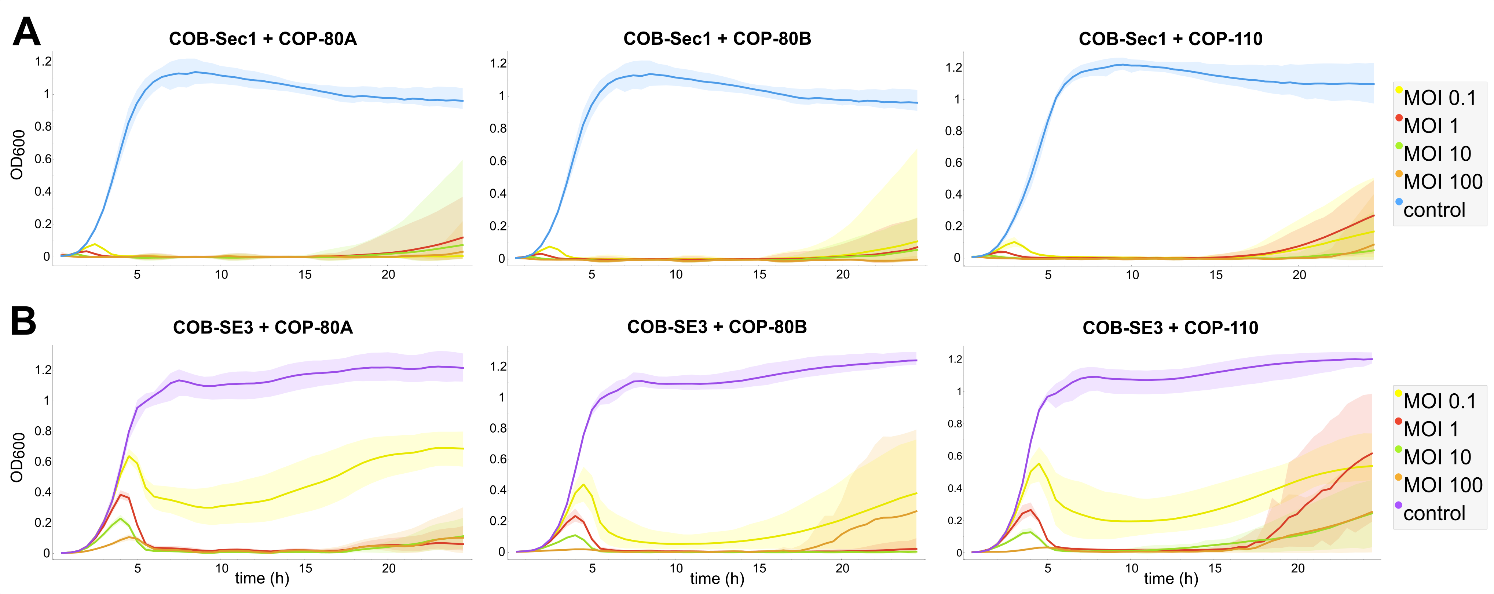


**Supplementary Figure 2.** **Antibacterial activity of phages COP-80A, COP-80B and COP-110 against strains COB-Sec1 (A) and COB-SE3 (B) at MOIs of 0.1, 1, 10 and 100 measured as growth kinetics (dynamic host range).**

Results of growth kinetics measurements are presented as mean values of two experiments (biological replicates) with a shaded area representing the range of measured OD_600_.


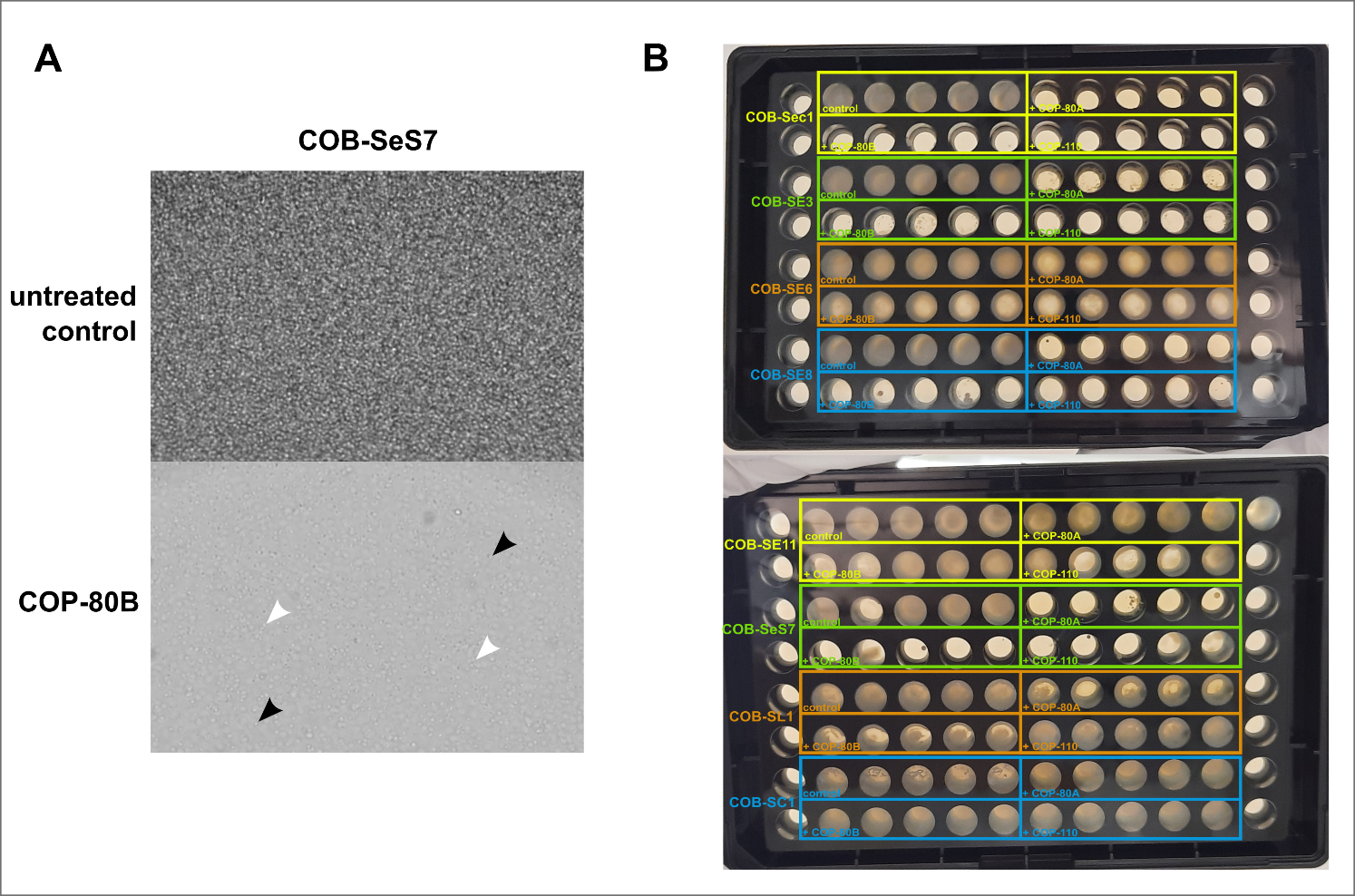


**Supplementary Figure 3: Antibiofilm activity of phages COP-80A, COP-80B and COP-110 against *S. epidermidis* (COB-Sec1, COB-SE3, COB-SE6, COB-SE8, COB-SE11), *S. pasteuri* (COB-SeS7), *S. lugdunensis* (COB-SL1) and *S. capitis* (COB-SC1) strains**.

Twenty-four hours old mature biofilms were challenged with phages for 24 h and then examined by brightfield microscopy (A) and visually by capturing the images of microtiter plates (B).

**(A)** Biofilm of *S. pasteuri* COB-SeS7 treated with phage COP-80B has been visualized with brightfield microscopy at 100× magnification. Single cells are indicated by black arrows and cell aggregates by white arrows.

**(B)** Image of the microtiter plate to obtain a visual estimate of the biofilm appearance. Each combination strain-phage was tested in five technical replicates; the tested phage-host combinations are indicated by text in different colors.

## Supplementary Tables

**Supplementary Table 1: Sources of sewage samples for isolation.**

**Supplementary Table 2: List of isolated phages.**

**Supplementary Table 3: Summary of quality trimmed read assembly procedure for all phages sequenced in this study.**

**Supplementary Table 4: Prophage contamination detected in all sequenced phages.**

**Supplementary Table 5. General genomic features of phages free from prophage contamination and their classification.**

**Supplementary Table 6. Indicators of temperate lifestyle: lifestyle prediction by PHACTS, presence of identifiable genes associated with lysogeny, presence of att sites (PHASTER), phage genome *blastn* against NCBI non redundant database.**

**Supplementary Table 7. Indicators of generalized transduction in sequenced phages.**

**Supplementary table 8. General genomic features of *Staphylococcus epidermidis* strains sequenced in this study.**

**Supplementary table 9. Analysis of contigs with elements associated with plasmids.**

**Supplementary table 10. Presence of temperate phages in genomes of *S. epidermidis* strains sequenced in this study determined by PHASTER.**

**Supplementary table 11. Currently known virulence factors of *S. epidermidis* detected in strains sequenced in this study identified by VFAnalyzer and *blastp*.**

**Supplementary table 12. Genetic determinants of antibiotic resistance in *S. epidermidis* identified by *blast*.**

**Supplementary table 13. Phage defense mechanisms in *S. epidermidis* strains sequenced in this study predicted by PADLOCK.**

**Supplementary table 14: Dynamic host range assay, OD600 of bacterial cultures after 24 h culture in the presence of phages COP-80A, COP-80B and COP-110 at different MOIs.**

**Supplementary table 15: Dynamic host range assay, duration of growth inhibition of bacterial strains COB-Sec1 and COB-SE3 in the presence of phages COP-80A, COP-80B and COP-110 at different MOIs.**
